# The Impact of Conception Method, Chorionicity, Amnionicity, Fetal Growth Types, and Birth Order on the Postnatal Status of Twins Born Vaginally and by Cesarean Section: A Retrospective Analysis of Data from a University Centre of Obstetrics and Gynecology (1990–2017)

**DOI:** 10.3390/jcm14207317

**Published:** 2025-10-16

**Authors:** Karolina Pełka, Sara Sawicka, Aleksandra Bator, Magdalena Wnęk, Jerzy Florjański

**Affiliations:** 1 Student Scientific Group of Obstetric and Gynecology, Wroclaw Medical University, 50-368 Wroclaw, Poland; sara.sawicka@student.umw.edu.pl (S.S.); aleksandra.bator@student.umw.edu.pl (A.B.); magdalena.wnek@student.umw.edu.pl (M.W.); 2 University Centre of Obstetrics and Gynecology, Wroclaw Medical University, 50-556 Wroclaw, Poland; jerzy.florjanski@umw.edu.pl

**Keywords:** twin pregnancy, type of twin growth, perinatal outcomes, chorionicity, amnionicity, ART, mode of delivery, Apgar score, fertilization in vitro

## Abstract

**Background/Objectives**: Twin pregnancies have long been of interest to the scientific community. Particular attention has been paid to factors influencing the postnatal condition of newborns. However, findings related to this issue, obtained in various centres, remain inconclusive. **Methods**: Data from 845 pairs of twins born between 1990 and 2017 at the University Centre of Obstetrics and Gynecology in the Wroclaw Medical University were analyzed. The postnatal condition was assessed based on the Apgar score at the 5th minute after birth. The Apgar scores were categorized into three groups: 8–10 indicated a good condition, 4–7 moderate, and 0–3 poor. Pregnancies with unknown chorionicity and amnionicity, monochorionic and monoamniotic pregnancies, still births, and cases with fetal defects were excluded from the study (126 cases). Finally, data of 719 pairs of twins were included. **Results**: Twins with a birth weight difference of less than 15% were more likely to receive a good Apgar score compared to those with a greater weight discrepancy (*p* < 0.001). The second-born twin was more likely to receive a good Apgar score compared to the first-born (*p* < 0.001). A higher proportion of twins delivered by cesarean section received a good Apgar score compared to those delivered vaginally (*p* < 0.001). The method of conception, chorionicity, amnionicity and being the smaller or bigger twin in the pair did not significantly affect the perinatal outcomes. **Conclusions**: Delivery method, birth order, and weight discrepancy play a key role in postnatal condition of twins, highlighting the importance of understanding these factors to optimize the management of twin pregnancies.

## 1. Introduction

Scientific advances have made it possible to effectively treat infertility. Although assisted reproductive methods (ART) are now common practice, there are still some concerns about their safety [1]. An important fact is that their application results in multiple pregnancies more often than after natural conception [2,3], due to the common practice of transferring two embryos. For this reason, single embryo transfer is currently recommended to reduce the incidence of twin pregnancies and related complications [4].

Twin pregnancies, which account for 2–3% of all pregnancies [5], are high-risk conditions that require specialized care [6]. Research on twin pregnancies is important because of the nine-times-higher incidence of complications compared to singleton pregnancies [4], such as pre-eclampsia, higher risk of cesarean section, and preterm delivery [7].

Twin pregnancies due to chorionicity and amnioticity are classified as DCDA (dichorionic, diamniotic), MCDA (monochorionic, diamniotic), and MCMA (monochorionic, monoamniotic) [8]. MCDA pregnancies, in which twins share a single placenta, are considered particularly prone to maternal–fetal complications, including fetal growth restriction (FGR). FGR has been defined as an estimated fetal weight less than the 10th percentile for gestational age as evaluated by prenatal ultrasound examination [9,10].

Studies comparing perinatal outcomes of twin pregnancies conceived naturally and by ART are inconsistent. In most of the available literature, the prevailing view is that there are no statistically significant differences in perinatal outcomes in newborns conceived through ART, although there are also reports of poorer perinatal outcomes (in both single and multiple pregnancies) [2,11].

Nevertheless, a twin pregnancy carries a higher risk for the mother and fetus than a single pregnancy, regardless of the method of conception [12]. At the same time, it should be emphasized that usually the second twin is characterized by a worse condition, as it remains in the uterus longer and is subject to potential adverse factors such as placental failure longer in case of complications [13].

For this reason, it is important to determine the most optimal way to manage the pregnancy and delivery in order to maximize the perinatal outcomes of both twins, especially the second one. In the study, the Apgar score was used as an indicator of the condition of the child immediately after birth. This scale was chosen because of its widespread use. Despite its limitations, it remains a good indicator for the initial assessment of the condition of a newborn and the early need for intervention. However, it should be noted that the lack of long-term data on twins makes it impossible to link perinatal outcomes with the further development of children.

In view of the aforementioned considerations, we decided to conduct a study aimed at comparing perinatal outcomes according to the method of conception, chorionicity, amnionicity, type of fetal growth and method of termination of pregnancy. The main objective of the study was to identify the parameters that primarily determine the condition of both twins, which may contribute to improving the management of twin pregnancies.

## 2. Materials and Methods

A retrospective cohort study was conducted using data from twin pregnancies and their newborns delivered at the University Centre of Obstetrics and Gynecology, Wroclaw Medical University, between 1990 and 2017. Information was collected from hospital medical records and included the following: parity (primiparaous or multiparaous), maternal age, gestational age, mode of conception (spontaneous or assisted reproduction), mode of delivery (vaginal or cesarean section) chorionicity, amnionicity, intertwin weight difference, and Apgar score at the fifth minute after birth. The primary dataset consisted of 845 pregnancies (1690 newborns). The analysis included only cases resulting in live births. Pregnancies with indeterminate chorionicity or amnionicity were excluded, as well as those affected by fetal chromosomal abnormalities, structural malformations, twin reversed arterial perfusion (TRAP), or twin-to-twin transfusion syndrome (TTTS) in monochorionic gestations (Table 1). Additionally, due to the small number (n = 7) of monochorionic monoamniotic pregnancies and the risk of unreliable comparisons, these cases were also excluded.

The final dataset consisted of 719 women with twin pregnancies, totaling 1438 neonates. Chorionicity and amnionicity were determined through first-trimester ultrasound examinations. Fetal growth discordance was assessed based on birth weight differences between co-twins and categorized into five groups: <15%, 15–19%, 20–24%, 25–30%, and >30% [14]. The percentage of weight discordance was calculated using the following formula: (weight of larger twin − weight of smaller twin)/weight of larger twin × 100%.

Neonatal condition was evaluated using the 5-minute Apgar score, recorded by the attending neonatologist according to standard clinical practice. For the purpose of analysis, Apgar scores were categorized into three groups: good (8–10 points), moderate (4–7 points), and poor (0–3 points).

No formal adjustment for gestational age or maternal factors was applied in the main analysis, as the study primarily aimed to describe general associations between perinatal factors and neonatal outcomes. However, supplementary analyses considering these variables were included in Appendix A (Table A1, Table A2, Table A3, Table A4, Table A5 and Table A6) to illustrate influences of gestational and maternal characteristics on the results.

Statistical comparisons between groups were performed using R (v4.3.2) and Python (v3.11). Pearson’s chi-square test was used for categorical variables, with statistical significance set at *p* < 0.05. The sample size (n) was defined as the number of individual newborns rather than twin pairs, as each neonate was assessed separately with an individual Apgar score. This approach allowed for a more accurate analysis of the relationship between perinatal factors (such as birth order, mode of delivery, or weight discordance) and neonatal outcomes, acknowledging the possibility of differing postnatal conditions within twin pairs.

## 3. Results

For analytical purposes, the study population was stratified into subgroups according to several perinatal factors: the mode of conception (spontaneous vs. in vitro fertilization), chorionicity and amnionicity (dichorionic diamniotic—DCDA and monochorionic diamniotic—MCDA), degree of fetal growth discordance (<15%, 15–19%, 20–24%, 25–30%, and >30%), being the smaller or bigger twin, type of delivery (vaginal birth vs. cesarean section), and birth sequence (first-born vs. second-born).

### 3.1. Conception Method

**Figure 1:**
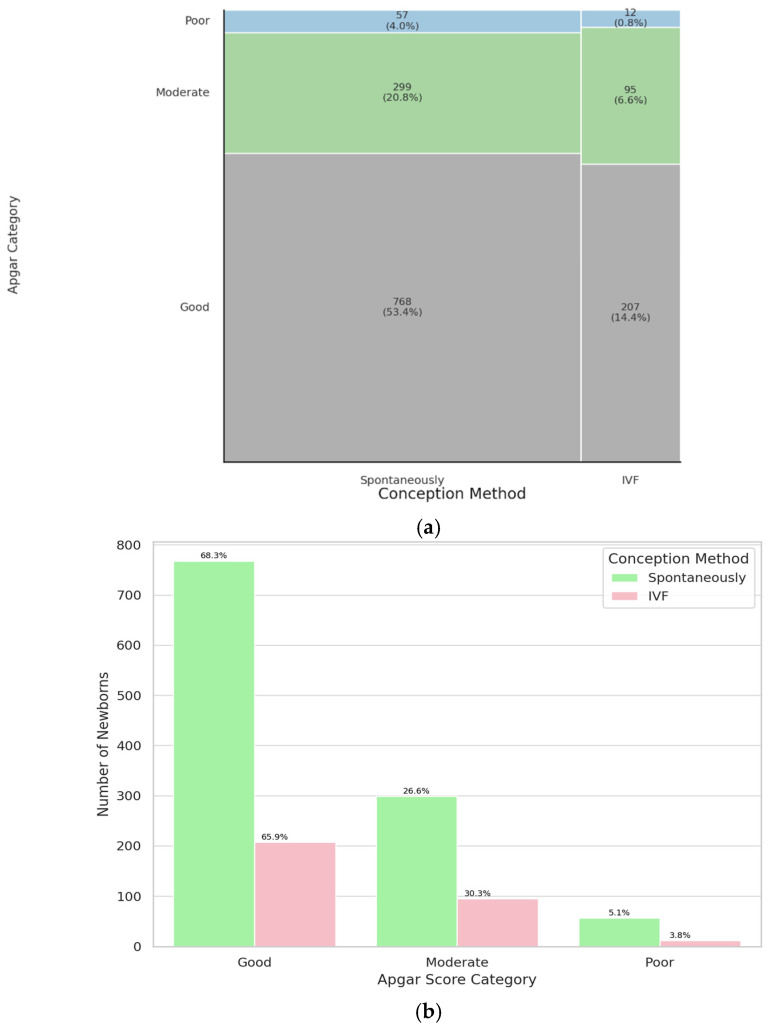
Mosaic (**a**) and bar (**b**) plot illustrating representation of twins conceived spontaneously and by IVF in a specific Apgar category.

To evaluate the impact of the conception method on neonatal condition, 5-minute Apgar scores were compared between twins conceived via in vitro fertilization (IVF) and those conceived naturally. The difference was not statistically significant (*p* = 0.33). The sample size for the IVF group was 314 newborns, and for the naturally conceived group, 1124 newborns. These findings suggest that the method of conception does not significantly influence the immediate postnatal condition of twins, as assessed by the 5-minute Apgar score. Results are shown on Figure 1. The study group was characterized in Table A1. In summary, no significant association was found between twins conceived by IVF and those conceived spontaneously.

### 3.2. Chorionicity and Amnionicity

**Figure 2:**
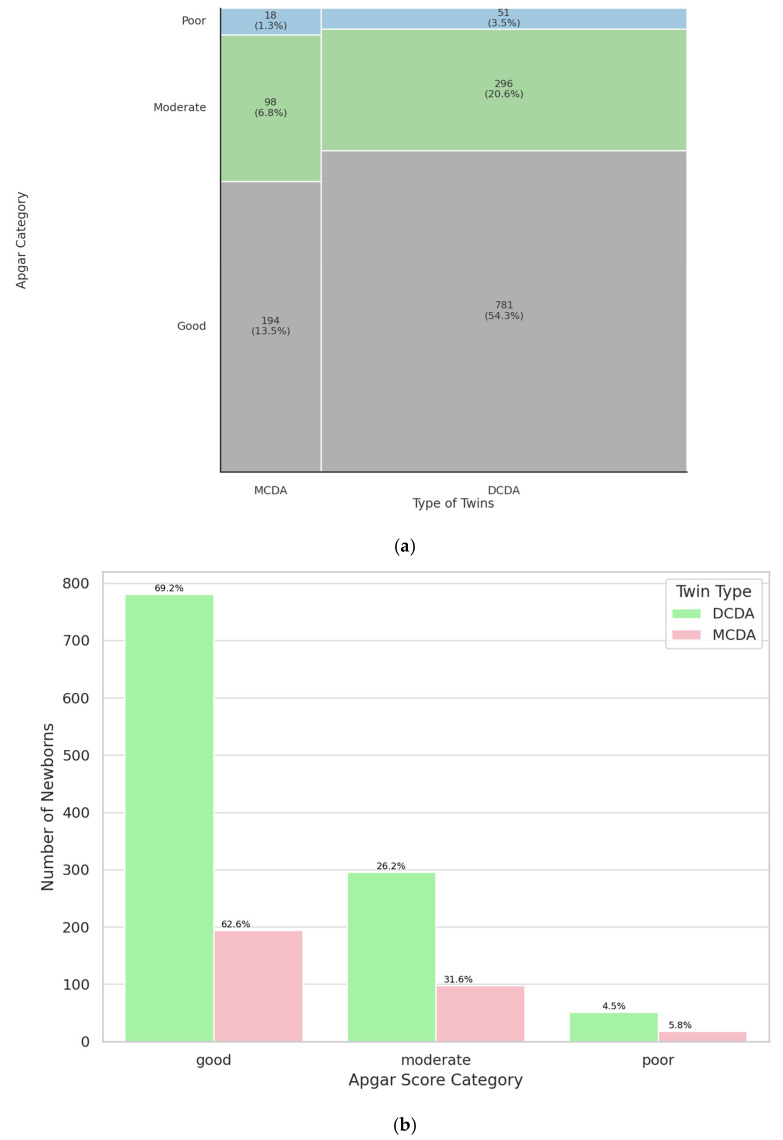
Mosaic (**a**) and bar (**b**) chart illustrating representation of MCDA and DCDA twins in a specific Apgar category.

To assess whether the chorionicity and amnionicity impacts neonatal status, 5-minute Apgar scores were compared between dichorionic diamniotic (DCDA) and monochorionic diamniotic (MCDA) twins. The difference was not statistically significant (*p* = 0.21). The sample size for the MCDA group was 310, and for the DCDA group 1128. These findings suggest that chorionicity does not significantly impact neonatal condition at 5-minutes postpartum, as measured by the Apgar score. Results are shown on Figure 2. The study group was characterized in Table A2. In summary, chorionicity and amnionicity were not associated with significant differences in 5-minute Apgar scores.

### 3.3. Weight Difference

#### 3.3.1. Inter-Twin Weight Discrepancy

**Figure 3:**
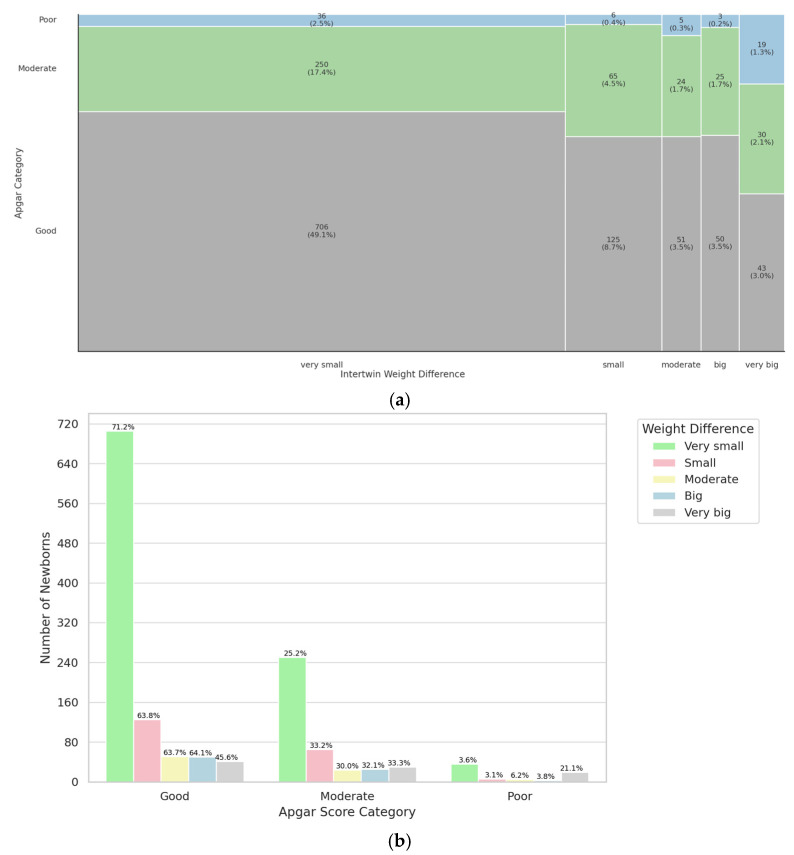
Mosaic (**a**) and bar (**b**) plot illustrating representation of twins with different growth discordance in a specific Apgar category.

The next parameter analyzed was the weight difference between twins. Twins were divided into five groups based on the percentage of difference in weight (very small difference: <15%, small difference: 15–19%, moderate difference: 20–24%, big difference: 25–30%, and very big difference: >30%). The difference was statistically significant (*p* < 0.001). Among twins with a good Apgar category there are proportionally more cases of very small weight difference. Results are shown on Figure 3. The study group was characterized in Table A3. In summary, the degree of birth weight discordance showed a significant association with the 5-minute Apgar score, with smaller differences corresponding to better neonatal outcomes.

#### 3.3.2. Being Bigger or Smaller Twin

**Figure 4:**
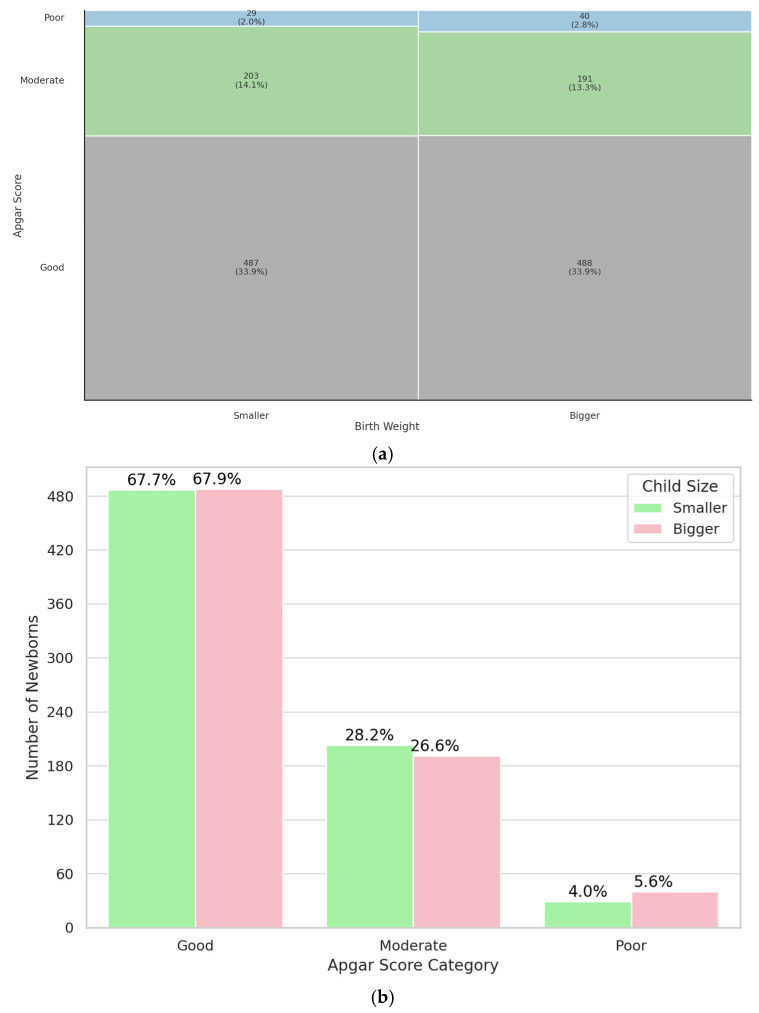
Mosaic (**a**) and bar (**b**) plot illustrate the representation of bigger and smaller newborn in a pair of twins in a specific Apgar category.

Subsequently, it was analyzed whether the smaller twin in a pair had a lower 5-minute Apgar score. Our findings suggest that being the smaller or larger twin within a pair does not significantly influence immediate postnatal condition, as assessed by the 5-minute Apgar score (*p* = 0.35). Results are shown on Figure 4. The study group was characterized in Table A4. Overall, no significant relationship was observed between being the smaller or larger twin and the 5-minute Apgar score.

### 3.4. Delivery Method

**Figure 5:**
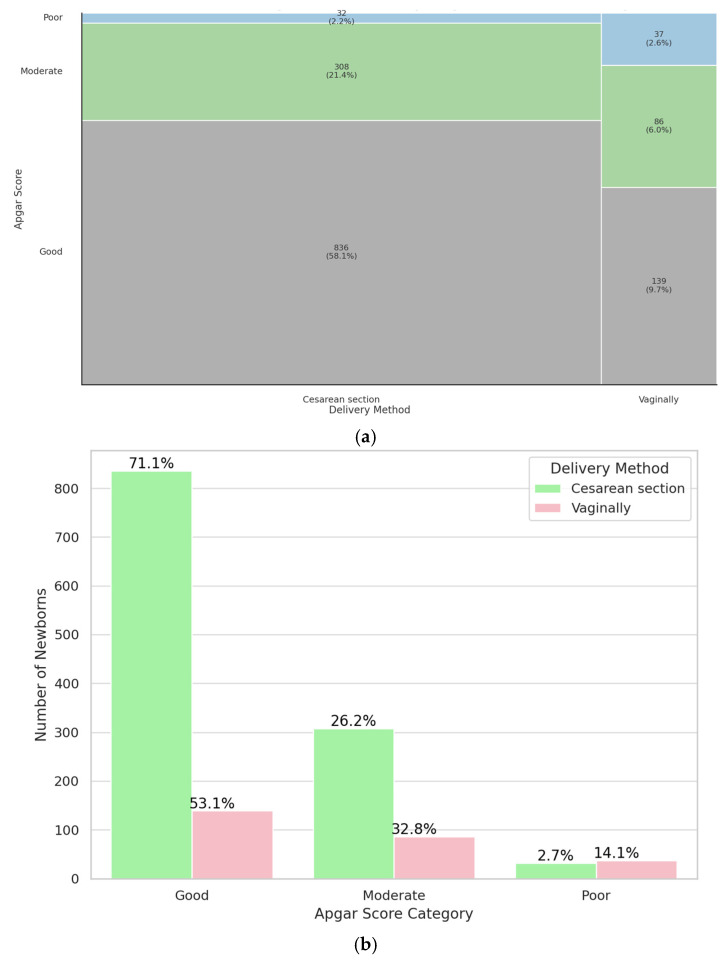
Mosaic (**a**) and bar (**b**) plot illustrating representation of twins delivered vaginally and by cesarean section in a specific Apgar category.

The influence of the mode of delivery on the immediate postnatal condition of twins was subsequently evaluated. The results showed a significant association (*p* < 0.001), indicating that twins delivered via cesarean section were proportionally more likely to have a good 5-minute Apgar score compared to those born vaginally. Among the study population, 1176 neonates were delivered by cesarean section, while 262 were born via vaginal delivery. These findings suggest that the mode of delivery plays a significant role in neonatal condition. Twins born by a cesarean section were more likely to receive a good Apgar score than twins born vaginally. Results are shown on Figure 5. The study group was characterized in Table A5. Collectively, the results indicate that cesarean section appears to provide more favorable immediate neonatal outcomes in twin deliveries.

### 3.5. Birth Order

**Figure 6:**
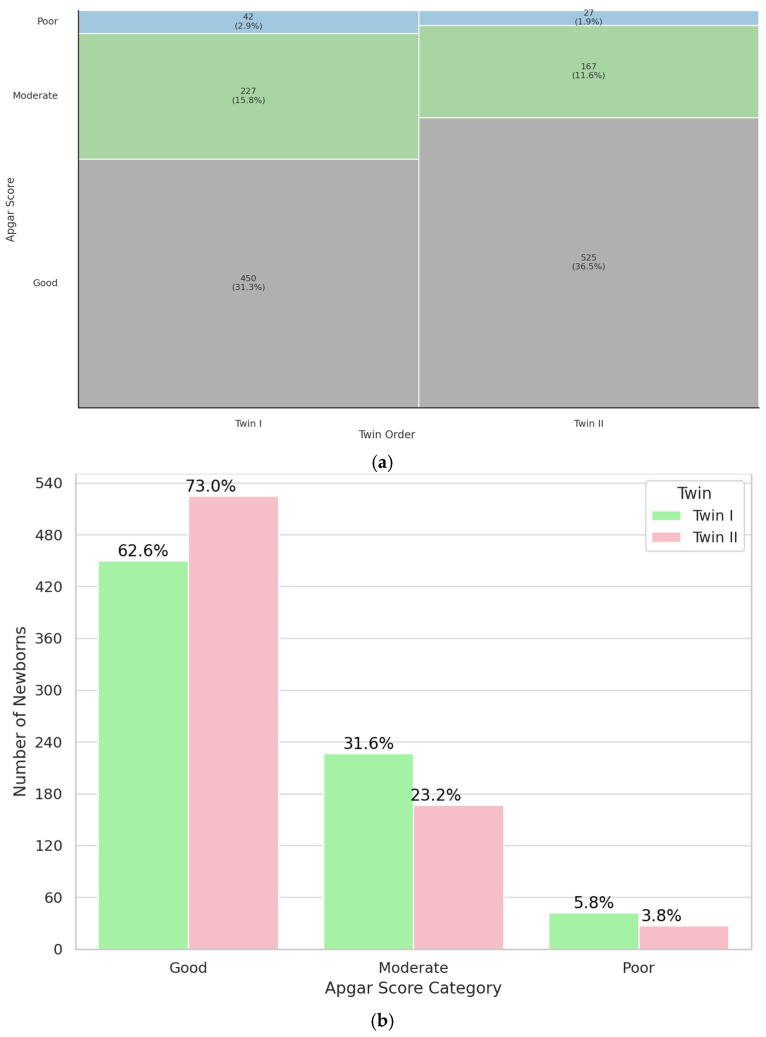
Mosaic (**a**) and bar (**b**) plot illustrating representation of twins born as a first and second in a specific Apgar category.

Birth order (Twin I vs. Twin II) was the final parameter evaluated for its influence on neonatal condition at 5-minute postpartum. The results revealed a significant association (*p* < 0.001), indicating that the second-born twin was more likely to have a good 5-minute Apgar score compared to the first-born twin. Results are shown on Figure 6. The study group was characterized in Table A6. Altogether, these findings demonstrate that birth order significantly affects the immediate postnatal condition, with the second-born twin more frequently achieving higher Apgar scores.

A more detailed analysis and summary of the results can be found in Appendix A.

## 4. Discussion

Our retrospective analysis of 1438 twins provides valuable insights into the impact of conception method, chorionicity, amnionicity, fetal growth discordance, birth order, and delivery mode on neonatal condition as assessed by the 5-minute Apgar score. Our findings indicate that the method of conception (IVF vs. natural), chorionicity (MCDA vs. DCDA), amnionicity and being bigger or smaller twin do not significantly impact neonatal outcomes as measured by the 5-minute Apgar score. However, significant associations were observed between neonatal outcomes and delivery method, birth order, and intertwin weight difference.

### 4.1. Conception Method

In our study, no statistically significant difference in 5-minute Apgar scores was observed between twins conceived via ART (n = 314) and those conceived spontaneously (n = 1124; *p* = 0.33). The majority of neonates in both groups achieved a “good” Apgar score (66% in ART vs. 68% in spontaneous conception). This finding is particularly significant in the context of the increasing prevalence of assisted reproductive technologies (ART) and requires thorough analysis in light of existing scientific reports. Our observation is consistent with findings from other studies, which indicate no significant differences in the immediate postpartum condition between twins conceived naturally and those conceived via IVF [2,15,16]. Also, Lin et al. analyzed the outcomes of 1270 pairs of twins and found no significant differences in Apgar scores between ART-conceived and naturally conceived twins. While ART twins had reduced odds of low birth weight (LBW), prematurity, and intrauterine growth restriction (IUGR), Apgar scores, neonatal mortality, and NICU admissions were comparable between the two groups [17]. Another study found that first-minute Apgar scores were similar between ART twins (6.21 ± 2.02) and spontaneous twins (6.30 ± 1.87), with no statistically significant difference (*p* = 0.383). Similarly, fifth-minute Apgar scores were comparable: 7.42 ± 2.11 for ART twins versus 8.12 ± 1.56 for spontaneous twins (*p* = 0.103) [18]. These findings contrast with several previous studies that reported a strong correlation between ART and increased perinatal risks [19,20].

Upon closer comparison, these discrepancies may be attributed to several methodological and demographic differences. For example, some earlier studies included broader perinatal outcomes from singleton and multiple pregnancies combined, or focused solely on high-risk subpopulations such as women over 40 or with underlying infertility diagnoses. In contrast, our study and others with similar findings specifically analyzed twin pregnancies, controlling for plurality, which is a known independent risk factor for adverse neonatal outcomes. Differences in sample size and statistical power may also influence the results. Our study included over 1400 twins, offering robust power to detect even modest differences, whereas some earlier studies were based on smaller cohorts or registry data without detailed clinical stratification. Moreover, temporal factors likely play a role. Studies reporting worse outcomes for ART twins were often conducted during earlier periods, when ART techniques were less refined. Recent data, including ours, reflect advancements such as elective single embryo transfer (eSET), improved embryo culture conditions, and better maternal monitoring, which have significantly improved perinatal outcomes in ART pregnancies [21,22].

The discrepancy may stem from advances in ART methodologies, including elective single embryo transfer, improved luteal phase support, and refined embryo culture techniques, which have been shown to mitigate risks historically associated with ART pregnancies [21,22]. Research conducted by Rashid et al. identified poorer neonatal outcomes in ART-conceived twins compared to those conceived spontaneously, including lower average birth weights and a higher number of neonates with Apgar scores below 7 at five minutes [23]. However, their study was conducted in a different healthcare context, where variations in perinatal care practices, ART protocols, and maternal characteristics such as average maternal BMI, age at conception, or prevalence of underlying health conditions may have influenced outcomes.

### 4.2. Type of Twins

There are no statistically significant differences in our study (*p* = 0.21) in Apgar scores between monochorionic diamniotic (MCDA) and dichorionic diamniotic (DCDA) twins. Our findings are consistent with those of a prospective cohort study by Coutinho Nunes et al., which reported no statistically significant differences in Apgar scores between MCDA and DCDA twins, despite variations in gestational age and birth weights [24]. Most studies suggest a higher risk of complications in monochorionic pregnancies. Several studies have reported an increased risk of low Apgar scores in MCDA twins compared to their DCDA counterparts. For instance, Rissanen et al. reported that MCDA twins had lower birth weights, lower Apgar scores, and a higher likelihood of requiring NICU admission compared to DCDA twins [25]. Similarly, Wandel et al. found a 1.7-fold increased risk of an Apgar score < 7 at five minutes in MCDA twins [26], while Miranda et al. observed a slightly greater reduction in fifth-minute Apgar scores among MCDA twins (0.7 vs. 0.6) [27]. These discrepancies may be partially explained by differences in study design and population characteristics. For example, while our cohort excluded complicated monochorionic pregnancies such as those affected by twin-to-twin transfusion syndrome (TTTS), some of the referenced studies included all MCDA twins regardless of complication status, which could have influenced the overall Apgar outcomes [25,26,27].

Additionally, the study by Rissanen et al. was based on nationwide registry data collected over several years in Finland, encompassing a heterogeneous population and clinical practices across various hospitals [25]. In contrast, our data were derived from a single-centre cohort with standardized perinatal care and chorionicity-specific management, which may have mitigated adverse outcomes in MCDA twins. The slightly lower Apgar scores reported by Miranda et al. [27] and Wandel et al. [26] were observed in retrospective cohorts over long time spans, during which clinical protocols and neonatal care practices may have evolved. This highlights the importance of considering both temporal and methodological variability when interpreting differences in neonatal outcomes by chorionicity.

### 4.3. Weight Difference

Our study further confirms the impact of birth weight discordance on neonatal outcomes, particularly Apgar scores. We observed a statistically significant difference (*p* < 0.001) in Apgar scores depending on the percentage of inter-twin birth weight discrepancy, with a higher proportion of twins with minimal weight discordance (≤15%) falling into the favorable Apgar category. This aligns with prior research, which has identified weight discordance as a risk factor for lower Apgar scores and increased NICU admissions, especially in premature twins. However, not all studies have found a uniform association. Appleton et al. reported no significant differences in Apgar scores when comparing concordant and discordant twin pairs in near-term pregnancies, although adverse outcomes were more pronounced when one twin was small for gestational age (SGA) [28]. Similarly, Florjański et al. highlighted that in MCDA pregnancies, the second twin with discordant growth exhibited significantly lower Apgar scores compared to its co-twin [29]. These findings suggest that while birth weight discordance alone may not always predict Apgar score deterioration, it becomes particularly relevant when combined with factors such as chorionicity and fetal growth abnormalities.

An often-overlooked aspect in the literature concerning weight discrepancies between twins involves the perinatal outcomes of the smaller versus the larger twin. Contrary to common assumptions, our data indicated that being the smaller twin does not correlate with poorer Apgar scores at the 5-minute mark post-birth. Most studies associate lower birth weight with a heightened risk of fetal and neonatal death [30,31,32,33]. Branum et al. found that mortality among smaller twins with significant weight discordance was 11 times higher compared to their non-discordant counterparts. Interestingly, both smaller and larger twins experiencing a ≥30% weight discrepancy displayed similarly increased mortality rates [34]. In contrast, Demissie et al. reported that stillbirth risk in both twins with birth weight discordance was increased [35]. This may support the view that discordance signals overall intrauterine stress rather than isolated fetal growth restriction.

### 4.4. Birth Order

The perinatal status of the second twin is of high interest to scientists. Unexpectedly, in our study population, the twin born second in the pair was more likely to have better perinatal outcomes at 5 min after birth than the first of the pair (*p* < 0.001). Unfortunately, no studies with similar results have been identified.

In the case of the results obtained, the explanation could be the fact that most deliveries were performed by cesarean section (81.8%), which in the case of second twins, gives better perinatal outcomes than termination of pregnancy by natural delivery. However, the results in Table A6 reveal that second twins had better outcomes regardless of the mode of delivery (*p* < 0.001 in both the vaginal delivery and CC rows). This indicates that there is a statistically significant difference in perinatal status between the groups studied, in benefit of the second twin. Nevertheless, the result is unclear. One of the limitations of the study is the lack of data on umbilical cord blood pH; perhaps the results would be different if this parameter were taken into account.

Usually, the status of the second twin is described as poorer compared to the first twin. A 2019 study by Florjanski et al. found that second twins were associated with worse postpartum outcomes when the pregnancy was terminated vaginally rather than by cesarean section [36]. An explanation could be the fact that the drop in umbilical artery blood pH in the second twin is faster in vaginal deliveries, so the time interval between the delivery of the first and second twin is crucial [37]. On the other hand, a 2024 study of 409 twin pregnancies by Rahman et al. showed that the interval between the vaginal delivery of both twins has no effect on the postpartum status of the second twin [38].

The problem of the interval between the birth of the first and second twins of vaginal delivery remains unresolved. In a study by Cukierman et al. (2019), second twins scored lower on the Apgar scale if the interval between the birth of both twins was >30 min [39]. Such a view is replicated by many researchers [40,41,42]. Another study proved that an interval of even more than 10 min between births results in a greater exposure to lower Apgar scale scores of the second twins [43]. Thus, there is a belief that there is a relationship between the birth interval of the two twins and the perinatal outcome of the second twin, while it remains debatable whether the interval is a direct cause of poorer outcomes [44,45].

Another approach focuses attention on the gestational age of the twins. Erdemoglu et al. (2003) found that gestational age is the main predictor of Apgar score in second twins, and the interval between births only provides a better estimate of the condition of the newborn [46]. In contrast, S. L. Mok and T. K. Lo (2022) [47] showed that the prolonged time between births of both twins by vaginal route increased the risk of needing a cesarean section for the second twin, but did not increase the risk of adverse perinatal outcomes. Adverse outcomes for the second twin after vaginal delivery were mainly due to prematurity [47].

Furthermore, some studies indicate a correlation between poor perinatal outcomes and low birth weight, regardless of whether it is a multiple pregnancy [48].

### 4.5. Delivery Method

Due to the higher stress on the second twin and the risk of achieving a lower Apgar score after the birth of the first twin, twin births by the vaginal route pose a significant challenge for obstetricians [39]. Studies emphasize that the risk to the second twin may be even greater in the presence of various additional factors, including preterm birth and fetal position [16,49]. Among other things, the route of delivery is chosen depending on the positioning of the fetuses, although it is not entirely clear whether presentation should actually determine the type of pregnancy termination [50].

R. R. Jhaveri and T. K Nadkarni (2016) [51] noted that vaginal delivery was safe for both twins in vertex presentation, while cesarean section was safe for non-vertex presentation of the first twin. However, an Apgar score of 7 or less occurred more than three times more often in the second twin after vaginal delivery compared to cesarean section [51]. In a study by C. Adam et al. (1991), on the other hand, there were no differences in perinatal outcomes after vaginal delivery versus cesarean section [52].

In light of the aforementioned, the topic of interest is not only the perinatal outcomes of the first and second twins but also the effect of the method of termination of pregnancy on the condition of both of them. According to our study, performing a cesarean section resulted in a significantly better (*p* < 0.001) Apgar score at 5 min after birth compared to vaginal delivery.

The risk of adverse perinatal outcomes in second twins increases with planned vaginal deliveries and prolonged birth intervals, as shown, among other things, in a study of 1542 pairs of twins by Armson et al. (2006) [53]. Also in a study by Ylilehto et al. (2017), in the case of planned vaginal delivery, the second twin of a dichorionic pregnancy had a 5-minute Apgar score < 7 compared to a planned cesarean section, while in the group of monochorionic pregnancies, there were no statistically significant differences between planned vaginal delivery and cesarean section [54].

At the same time, this should not exclude the possibility of vaginal delivery, especially in twin pregnancies with vertex–vertex presentation [55], as there are also reports of no effect on primary outcome with planned cesarean section compared to planned vaginal delivery [56,57].

Therefore, there are reports in the literature about the non-impact of the method of termination of pregnancy on the postpartum condition; nevertheless, these studies are too limited to completely exclude the disadvantage of one of the solutions [58].

## 5. Conclusions

Determining the optimal care strategy in twin pregnancies is still contradictory and a topic of interest to many researchers. Evidence presented in this study shows that delivery method, birth order, and weight discordance play a key role in postnatal outcomes. On the contrary, conception method, chorionicity, and amnionicity do not significantly influence neonatal outcomes. However, it should also be noted that this study is limited by retrospective design, and the lack of long-term follow-up which prevents the evaluation of later health and developmental outcomes of twins. Another limitation is that the findings are based on the Apgar score which, while widely used, may be considered less objective than biochemical indicators such as umbilical cord blood pH. Future research with more extended follow-up is needed to better understand those factors in order to optimize the perinatal care in twin pregnancies.

## Figures and Tables

**Table 1:** Reason and number of excluded cases.

Reason for Exclusion	N (Newborns)
Unknown chorionicity/amnionicity	6
MCMA	7
TTTS	38
TRAP	1
Fetal defects (structural/chromosomal)	50

## Data Availability

The original contributions presented in this study are included in the article, Further inquiries can be directed to the corresponding author.

## Abbreviations

The following abbreviations are used in this manuscript:

ART	Assisted reproductive methods
DCDA	Dichorionic, diamniotic
MCDA	Monochorionic, diamniotic
MCMA	Monochorionic, monoamniotic
FGR	Fetal growth restriction
IVF	In vitro fertilization
LBW	Low birth weight
IUGR	Intrauterine growth restriction
TRAP	Twin reversed arterial perfusion
TTTS	Twin-to-twin transfusion syndrome
NICU	Neonatal Intensive Care Unit
SGA	Small for gestational age
MA	Maternal age
GA	Gestational age (in the moment of delivery)

## Appendix A

The attached tables present detailed data on the groups studied according to selected parameters. Due to their size, they have been included in Appendix A.

**Table A1:** Characteristics of the study group by method of conception and Apgar score.

Variable	Group Size (Percentage)	Age—Mean (Standard Deviation)	IVF			Spontaneously			*p*-Value
			Apgar 0–3	Apgar 4–7	Apgar 8–10	Apgar 0–3	Apgar 4–7	Apgar 8–10	
**Total**	1438 (100.0%)	29.5, 5.0	12 (0.8%)	95 (6.6%)	207 (14.4%)	57 (4.0%)	299 (20.8%)	768 (53.4%)	NS
**Primiparae**	834 (58.0%)	28.5, 4.9	10 (1.2%)	76 (9.1%)	164 (19.7%)	33 (4.0%)	153 (18.3%)	398 (47.7%)	NS
**Multiparae**	604 (42.0%)	30.8, 5.0	2 (0.3%)	19 (3.1%)	43 (7.1%)	24 (4.0%)	146 (24.2%)	370 (61.3%)	NS
**MA 18–24**	246 (17.1%)	22.5, 1.7	0 (0.0%)	1 (0.4%)	15 (6.1%)	15 (6.1%)	59 (24.0%)	156 (63.4%)	NS
**MA 25–34**	962 (66.9%)	29.3, 2.8	9 (0.9%)	80 (8.3%)	157 (16.3%)	35 (3.6%)	193 (20.1%)	488 (50.7%)	NS
**MA > 34**	230 (16.0%)	37.5, 2.4	3 (1.3%)	14 (6.1%)	35 (15.2%)	7 (3.0%)	47 (20.4%)	124 (53.9%)	NS
**GA < 28 weeks**	38 (2.6%)	27.5, 3.3	2 (5.3%)	6 (15.8%)	0 (0.0%)	14 (36.8%)	16 (42.1%)	0 (0.0%)	NS
**GA 28–32 weeks**	208 (14.5%)	29.5, 5.5	7 (3.4%)	25 (12.0%)	14 (6.7%)	21 (10.1%)	94 (45.2%)	47 (22.6%)	NS
**GA 33–37 weeks**	886 (61.6%)	29.5, 4.8	3 (0.3%)	55 (6.2%)	150 (16.9%)	15 (1.7%)	141 (15.9%)	522 (58.9%)	NS
**GA 38–43 weeks**	306 (21.3%)	29.5, 5.5	0 (0.0%)	9 (2.9%)	43 (14.1%)	7 (2.3%)	48 (15.7%)	199 (65.0%)	NS
**Twin I**	719 (50.0%)	29.5, 5.0	7 (1.0%)	55 (7.6%)	95 (13.2%)	35 (4.9%)	172 (23.9%)	355 (49.4%)	NS
**Twin II**	719 (50.0%)	29.5, 5.0	5 (0.7%)	40 (5.6%)	112 (15.6%)	22 (3.1%)	127 (17.7%)	413 (57.4%)	NS
**Vaginal delivery**	262 (18.2%)	28.9, 5.3	0 (0.0%)	1 (0.4%)	9 (3.4%)	37 (14.1%)	85 (32.4%)	130 (49.6%)	NS
**Cesarean section**	1176 (81.8%)	29.6, 5.0	12 (1.0%)	94 (8.0%)	198 (16.8%)	20 (1.7%)	214 (18.2%)	638 (54.3%)	<0.05
**MCDA**	310 (21.6%)	28.9, 5.4	3 (1.0%)	8 (2.6%)	13 (4.2%)	15 (4.8%)	90 (29.0%)	181 (58.4%)	NS
**DCDA**	1128 (78.4%)	29.6, 4.9	9 (0.8%)	87 (7.7%)	194 (17.2%)	42 (3.7%)	209 (18.5%)	587 (52.0%)	NS
**Weight difference < 15%**	992 (69.0%)	29.5, 5.1	8 (0.8%)	149 (15.0%)	149 (15.0%)	28 (2.8%)	191 (19.3%)	557 (56.1%)	NS
**Weight difference** **15–19%**	196 (13.6%)	29.2, 4.7	0 (0.0%)	19 (9.7%)	19 (9.7%)	6 (3.1%)	46 (23.5%)	106 (54.1%)	<0.05
**Weight difference** **20–24%**	80 (5.6%)	29.9, 5.6	2 (2.5%)	6 (7.5%)	12 (15.0%)	3 (3.8%)	18 (22.5%)	39 (48.8%)	NS
**Weight difference** **25–30%**	78 (5.4%)	29.9, 5.0	0 (0.0%)	4 (5.1%)	12 (15.4%)	3 (3.8%)	21 (26.9%)	38 (48.7%)	NS
**Weight difference** **>30%**	92 (6.4%)	29.0, 4.4	2 (2.2%)	7 (7.6%)	15 (16.3%)	17 (18.5%)	23 (25.0%)	28 (30.4%)	NS
**Smaller twin**	719 (50.0%)	29.5, 5.0	9 (1.3%)	42 (5.8%)	106 (14.7%)	31 (4.3%)	149 (20.7%)	382 (53.1%)	NS
**Bigger twin**	719 (50.0%)	29.5, 5.0	3 (0.4%)	53 (7.4%)	101 (14.0%)	26 (3.6%)	386 (53.7%)	386 (53.7%)	NS

**Table A2:** Characteristics of the study group by type of twins (MCDA vs. DCDA) and Apgar score.

Variable	Group Size (Percentage)	Age—Mean (Standard Deviation)	MCDA			DCDA			*p*-Value
			Apgar 0–3	Apgar 4–7	Apgar 8–10	Apgar 0–3	Apgar 4–7	Apgar 8–10	NS
**Total**	1438 (100.0%)	29.5, 5.0	18 (1.3%)	98 (6.8%)	194 (13.5%)	51 (3.5%)	296 (20.6%)	781 (54.3%)	NS
**Primiparae**	834 (58.0%)	28.5, 4.9	9 (1.1%)	68 (8.2%)	107 (12.8%)	34 (4.1%)	161 (19.3%)	455 (54.6%)	<0.01
**Multiparae**	604 (42.0%)	30.8, 5.0	9 (1.5%)	30 (5.0%)	87 (14.4%)	17 (2.8%)	135 (22.4%)	326 (54.0%)	NS
**MA 18–24**	246 (17.1%)	22.5, 1.7	6 (2.4%)	18 (7.3%)	46 (18.7%)	9 (3.7%)	42 (17.1%)	125 (50.8%)	NS
**MA 25–34**	962 (66.9%)	29.3, 2.8	7 (0.7%)	73 (7.6%)	114 (11.9%)	37 (3.8%)	200 (20.8%)	531 (55.2%)	<0.01
**MA > 34**	230 (16.0%)	37.5, 2.4	5 (2.2%)	7 (3.0%)	34 (14.8%)	5 (2.2%)	54 (23.5%)	125 (54.3%)	<0.05
**GA < 28 weeks**	38 (2.6%)	27.5, 3.3	2 (5.3%)	4 (10.5%)	0 (0.0%)	14 (36.8%)	18 (47.4%)	0 (0.0%)	NS
**GA 28–32 weeks**	208 (14.5%)	29.5, 5.5	7 (3.4%)	20 (9.6%)	15 (7.2%)	21 (10.1%)	99 (47.6%)	46 (22.1%)	NS
**GA 33–37 weeks**	886 (61.6%)	29.5, 4.8	6 (0.7%)	62 (7.0%)	130 (14.7%)	12 (1.4%)	134 (15.1%)	542 (61.2%)	<0.001
**GA 38–43 weeks**	306 (21.3%)	29.5, 5.5	3 (1.0%)	12 (3.9%)	49 (16.0%)	4 (1.3%)	45 (14.7%)	193 (63.1%)	NS
**Twin I**	719 (50.0%)	29.5, 5.0	12 (1.7%)	56 (7.8%)	87 (12.1%)	30 (4.2%)	363 (50.5%)	363 (50.5%)	NS
**Twin II**	719 (50.0%)	29.5, 5.0	6 (0.8%)	42 (5.8%)	107 (14.9%)	21 (2.9%)	125 (17.4%)	418 (58.1%)	NS
**Vaginal delivery**	262 (18.2%)	28.9, 5.3	9 (3.4%)	22 (8.4%)	21 (8.0%)	28 (10.7%)	64 (24.4%)	118 (45.0%)	NS
**Cesarean section**	1176 (81.8%)	29.6, 5.0	9 (0.8%)	76 (6.5%)	173 (14.7%)	23 (2.0%)	232 (19.7%)	663 (56.4%)	NS
**IVF**	314 (21.8%)	30.7, 4.2	3 (1.0%)	8 (2.5%)	13 (4.1%)	9 (2.9%)	87 (27.7%)	194 (61.8%)	NS
**Spontaneous conception**	1124 (78.2%)	29.1, 5.2	15 (1.3%)	90 (8.0%)	181 (16.1%)	42 (3.7%)	209 (18.6%)	587 (52.2%)	NS
**Weight difference < 15%**	992 (69.0%)	29.5, 5.1	5 (0.5%)	37 (3.7%)	126 (12.7%)	31 (3.1%)	213 (21.5%)	580 (58.5%)	NS
**Weight difference** **15–19%**	196 (13.6%)	29.2, 4.7	2 (1.0%)	28 (14.3%)	36 (18.4%)	4 (2.0%)	37 (18.9%)	89 (45.4%)	NS
**Weight difference** **20–24%**	80 (5.6%)	29.9, 5.6	2 (2.5%)	7 (8.8%)	9 (11.2%)	3 (3.8%)	42 (52.5%)	42 (52.5%)	NS
**Weight difference** **25–30%**	78 (5.4%)	29.9, 5.0	1 (1.3%)	11 (14.1%)	14 (17.9%)	2 (2.6%)	14 (17.9%)	36 (46.2%)	NS
**Weight difference** **>30%**	92 (6.4%)	29.0, 4.4	8 (8.7%)	15 (16.3%)	9 (9.8%)	11 (12.0%)	15 (16.3%)	34 (37.0%)	<0.05
**Smaller twin**	719 (50.0%)	29.5, 5.0	9 (1.3%)	48 (6.7%)	98 (13.6%)	31 (4.3%)	143 (19.9%)	390 (54.2%)	NS
**Bigger twin**	719 (50.0%)	29.5, 5.0	9 (1.3%)	50 (7.0%)	96 (13.4%)	20 (2.8%)	153 (21.3%)	391 (54.4%)	NS

**Table A3:** Characteristics of the study group by different growth discordance and Apgar score.

Variable	Group Size (Percentage)	Age—Mean (Standard Deviation)	Very Small Difference <15%			Small Difference 15–19%			Moderete Difference 20–24%			Big Difference 25–30%			Very Big Difference >30%			*p*-Value
			Apgar 0–3	Apgar 4–7	Apgar 8–10	Apgar 0–3	Apgar 4–7	Apgar 8–10	Apgar 0–3	Apgar 4–7	Apgar 8–10	Apgar 0–3	Apgar 4–7	Apgar 8–10	Apgar 0–3	Apgar 4–7	Apgar 8–10	
**Total**	1438 (100.0%)	29.5, 5.0	36 (2.5%)	250 (17.4%)	706 (49.1%)	6 (0.4%)	65 (4.5%)	125 (8.7%)	5 (0.3%)	24 (1.7%)	51 (3.5%)	3 (0.2%)	25 (1.7%)	50 (3.5%)	19 (1.3%)	30 (2.1%)	43 (3.0%)	<0.001
**Primiparae**	834 (58.0%)	28.5, 4.9	23 (2.8%)	136 (16.3%)	399 (47.8%)	4 (0.5%)	45 (5.4%)	73 (8.8%)	5 (0.6%)	12 (1.4%)	39 (4.7%)	1 (0.1%)	19 (2.3%)	20 (2.4%)	10 (1.2%)	17 (2.0%)	31 (3.7%)	<0.001
**Multiparae**	604 (42.0%)	30.8, 5.0	13 (2.2%)	114 (18.9%)	307 (50.8%)	2 (0.3%)	20 (3.3%)	52 (8.6%)	0 (0.0%)	12 (2.0%)	12 (2.0%)	2 (0.3%)	6 (1.0%)	30 (5.0%)	9 (1.5%)	13 (2.2%)	12 (2.0%)	<0.001
**MA 18–24**	246 (17.1%)	22.5, 1.7	4 (1.6%)	45 (18.3%)	125 (50.8%)	2 (0.8%)	7 (2.8%)	27 (11.0%)	3 (1.2%)	4 (1.6%)	11 (4.5%)	1 (0.4%)	3 (1.2%)	4 (1.6%)	5 (2.0%)	1 (0.4%)	4 (1.6%)	<0.001
**MA 25–34**	962 (66.9%)	29.3, 2.8	27 (2.8%)	166 (17.3%)	461 (47.9%)	4 (0.4%)	48 (5.0%)	82 (8.5%)	0 (0.0%)	13 (1.4%)	29 (3.0%)	2 (0.2%)	20 (2.1%)	40 (4.2%)	11 (1.1%)	26 (2.7%)	33 (3.4%)	<0.001
**MA > 34**	230 (16.0%)	37.5, 2.4	5 (2.2%)	39 (17.0%)	120 (52.2%)	0 (0.0%)	10 (4.3%)	16 (7.0%)	2 (0.9%)	7 (3.0%)	11 (4.8%)	0 (0.0%)	2 (0.9%)	6 (2.6%)	3 (1.3%)	3 (1.3%)	6 (2.6%)	<0.05
**GA < 28 weeks**	38 (2.6%)	27.5, 3.3	7 (18.4%)	15 (39.5%)	0 (0.0%)	6 (15.8%)	4 (10.5%)	0 (0.0%)	0 (0.0%)	0 (0.0%)	0 (0.0%)	0 (0.0%)	2 (5.3%)	0 (0.0%)	3 (7.9%)	1 (2.6%)	0 (0.0%)	NS
**GA 28–32 weeks**	208 (14.5%)	29.5, 5.5	19 (9.1%)	80 (38.5%)	39 (18.8%)	0 (0.0%)	16 (7.7%)	6 (2.9%)	1 (0.5%)	6 (2.9%)	1 (0.5%)	3 (1.4%)	11 (5.3%)	8 (3.8%)	7 (3.4%)	6 (2.9%)	5 (2.4%)	NS
**GA 33–37 weeks**	886 (61.6%)	29.5, 4.8	8 (0.9%)	123 (13.9%)	483 (54.5%)	0 (0.0%)	35 (4.0%)	99 (11.2%)	4 (0.5%)	9 (1.0%)	35 (4.0%)	0 (0.0%)	8 (0.9%)	28 (3.2%)	6 (0.7%)	21 (2.4%)	27 (3.0%)	<0.001
**GA 38–43 weeks**	306 (21.3%)	29.5, 5.5	2 (0.7%)	32 (10.5%)	184 (60.1%)	0 (0.0%)	10 (3.3%)	20 (6.5%)	0 (0.0%)	9 (2.9%)	15 (4.9%)	0 (0.0%)	4 (1.3%)	14 (4.6%)	5 (1.6%)	2 (0.7%)	9 (2.9%)	<0.001
**Twin I**	719 (50.0%)	29.5, 5.0	24 (3.3%)	147 (20.4%)	325 (45.2%)	3 (0.4%)	40 (5.6%)	55 (7.6%)	3 (0.4%)	12 (1.7%)	25 (3.5%)	2 (0.3%)	11 (1.5%)	26 (3.6%)	10 (1.4%)	17 (2.4%)	19 (2.6%)	<0.001
**Twin II**	719 (50.0%)	29.5, 5.0	12 (1.7%)	103 (14.3%)	381 (53.0%)	3 (0.4%)	25 (3.5%)	70 (9.7%)	2 (0.3%)	12 (1.7%)	26 (3.6%)	1 (0.1%)	14 (1.9%)	24 (3.3%)	9 (1.3%)	13 (1.8%)	24 (3.3%)	<0.001
**Vaginal delivery**	262 (18.2%)	28.9, 5.3	19 (7.3%)	62 (23.7%)	105 (40.1%)	6 (2.3%)	8 (3.1%)	14 (5.3%)	2 (0.8%)	11 (4.2%)	9 (3.4%)	2 (0.8%)	4 (1.5%)	6 (2.3%)	8 (3.1%)	1 (0.4%)	5 (1.9%)	<0.001
**Cesarean section**	1176 (81.8%)	29.6, 5.0	17 (1.4%)	188 (16.0%)	601 (51.1%)	0 (0.0%)	57 (4.8%)	111 (9.4%)	3 (0.3%)	13 (1.1%)	42 (3.6%)	1 (0.1%)	21 (1.8%)	44 (3.7%)	11 (0.9%)	29 (2.5%)	38 (3.2%)	<0.001
**IVF**	314 (21.8%)	30.7, 4.2	8 (2.5%)	59 (18.8%)	149 (47.5%)	0 (0.0%)	19 (6.1%)	19 (6.1%)	2 (0.6%)	6 (1.9%)	12 (3.8%)	0 (0.0%)	4 (1.3%)	12 (3.8%)	2 (0.6%)	7 (2.2%)	15 (4.8%)	NS
**Spontaneous conception**	1124 (78.2%)	29.1, 5.2	28 (2.5%)	191 (17.0%)	557 (49.6%)	6 (0.5%)	46 (4.1%)	106 (9.4%)	3 (0.3%)	18 (1.6%)	39 (3.5%)	3 (0.3%)	21 (1.9%)	38 (3.4%)	17 (1.5%)	23 (2.0%)	28 (2.5%)	<0.001
**MCDA**	310 (21.6%)	28.9, 5.4	5 (1.6%)	37 (11.9%)	126 (40.6%)	2 (0.6%)	28 (9.0%)	36 (11.6%)	2 (0.6%)	7 (2.3%)	9 (2.9%)	1 (0.3%)	11 (3.5%)	14 (4.5%)	8 (2.6%)	15 (4.8%)	9 (2.9%)	<0.001
**DCDA**	1128 (78.4%)	29.6, 4.9	31 (2.7%)	213 (18.9%)	580 (51.4%)	4 (0.4%)	37 (3.3%)	89 (7.9%)	3 (0.3%)	17 (1.5%)	42 (3.7%)	2 (0.2%)	14 (1.2%)	36 (3.2%)	11 (1.0%)	15 (1.3%)	34 (3.0%)	<0.001
**Smaller twin**	719 (50.0%)	29.5, 5.0	20 (2.8%)	124 (17.2%)	352 (49.0%)	4 (0.6%)	30 (4.2%)	64 (8.9%)	3 (0.4%)	9 (1.3%)	28 (3.9%)	2 (0.3%)	13 (1.8%)	24 (3.3%)	11 (1.5%)	15 (2.1%)	20 (2.8%)	<0.001
**Bigger twin**	719 (50.0%)	29.5, 5.0	16 (2.2%)	126 (17.5%)	354 (49.2%)	2 (0.3%)	35 (4.9%)	61 (8.5%)	2 (0.3%)	15 (2.1%)	23 (3.2%)	1 (0.1%)	12 (1.7%)	26 (3.6%)	8 (1.1%)	15 (2.1%)	23 (3.2%)	<0.001

**Table A4:** Characteristics of the study group by being bigger or smaller twin and Apgar score.

Variable	Group Size (Percentage)	Age—Mean (Standard Deviation)	Smaller Twin			Bigger Twin			*p*-Value
			Apgar 0–3	Apgar 4–7	Apgar 8–10	Apgar 0–3	Apgar 4–7	Apgar 8–10	
**Total**	1438 (100.0%)	29.5, 5.0	40 (2.8%)	191 (13.3%)	488 (33.9%)	29 (2.0%)	203 (14.1%)	487 (33.9%)	NS
**Primiparae**	834 (58.0%)	28.5, 4.9	24 (2.9%)	109 (13.1%)	284 (34.1%)	19 (2.3%)	120 (14.4%)	278 (33.3%)	NS
**Multiparae**	604 (42.0%)	30.8, 5.0	16 (2.6%)	82 (13.6%)	204 (33.8%)	10 (1.7%)	83 (13.7%)	209 (34.6%)	NS
**MA 18–24**	246 (17.1%)	22.5, 1.7	8 (3.3%)	29 (11.8%)	86 (35.0%)	7 (2.8%)	31 (12.6%)	85 (34.6%)	NS
**MA 25–34**	962 (66.9%)	29.3, 2.8	27 (2.8%)	131 (13.6%)	323 (33.6%)	17 (1.8%	142 (14.8%)	322 (33.5%)	NS
**MA > 34**	230 (16.0%)	37.5, 2.4	5 (2.2%)	31 (13.5%)	79 (34.3%)	5 (2.2%)	30 (13.0%)	80 (34.8%)	NS
**GA < 28 weeks**	38 (2.6%)	27.5, 3.3	10 (26.3%)	9 (23.7%)	0 (0.0%)	6 (15.8%)	13 (34.2%)	0 (0.0%)	NS
**GA 28–32 weeks**	208 (14.5%)	29.5, 5.5	17 (8.2%)	59 (28.4%)	28 (13.5%)	11 (5.3%)	60 (28.8%)	33 (15.9%)	NS
**GA 33–37 weeks**	886 (61.6%)	29.5, 4.8	11 (1.2%)	93 (10.5%)	339 (38.3%)	7 (0.8%)	103 (11.6%)	333 (37.6%)	NS
**GA 38–43 weeks**	306 (21.3%)	29.5, 5.5	2 (0.7%)	30 (9.8%)	121 (39.5%)	5 (1.6%)	27 (8.8%)	121 (39.5%)	NS
**Twin I**	719 (50.0%)	29.5, 5.0	20 (2.8%)	90 (12.5%)	170 (23.6%)	22 (3.1%)	137 (19.1%)	280 (38.9%)	NS
**Twin II**	719 (50.0%)	29.5, 5.0	20 (2.8%)	101 (14.0%)	318 (44.2%)	7 (1.0%)	66 (9.2%)	207 (28.8%)	NS
**Vaginal delivery**	262 (18.2%)	28.9, 5.3	19 (7.3%)	45 (17.2%)	67 (25.6%)	18 (6.9%)	41 (15.6%)	72 (27.5%)	NS
**Cesarean Section**	1176 (81.8%)	29.6, 5.0	21 (1.8%)	146 (12.4%)	421 (35.8%)	11 (0.9%)	162 (13.8%)	415 (35.3%)	NS
**MCDA**	310 (21.6%)	28.9, 5.4	9 (2.9%)	48 (15.5%)	98 (31.6%)	9 (2.9%)	50 (16.1%)	96 (31.0%)	NS
**DCDA**	1128 (78.4%)	29.6, 4.9	31 (2.7%)	143 (12.7%)	390 (34.6%)	20 (1.8%)	153 (13.6%)	391 (34.7%)	NS
**Weight difference < 15%**	992 (69.0%)	29.5, 5.1	20 (2.0%)	124 (12.5%)	352 (35.5%)	16 (1.6%)	126 (12.7%)	354 (35.7%)	NS
**Weight difference** **15–19%**	196 (13.6%)	29.2, 4.7	4 (2.0%)	30 (15.3%)	64 (32.7%)	2 (1.0%)	35 (17.9%)	61 (31.1%)	NS
**Weight difference** **20–24%**	80 (5.6%)	29.9, 5.6	3 (3.8%)	9 (11.2%)	28 (35.0%)	2 (2.5%)	15 (18.8%)	23 (28.7%)	NS
**Weight difference** **25–30%**	78 (5.4%)	29.9, 5.0	2 (2.6%)	13 (16.7%)	24 (30.8%)	1 (1.3%)	12 (15.4%)	26 (33.3%)	NS
**Weight difference** **>30%**	92 (6.4%)	29.0, 4.4	11 (12.0%)	15 (16.3%)	20 (21.7%)	8 (8.7%)	15 (16.3%)	23 (25.0%)	NS
**IVF**	314 (21.8%)	30.7, 4.2	9 (2.9%)	42 (13.4%)	106 (33.8%)	3 (1.0%)	53 (16.9%)	101 (32.2%)	NS
**Spontaneous conception**	1124 (78.2%)	29.1, 5.2	31 (2.8%)	149 (13.3%)	382 (34.0%)	26 (2.3%)	150 (13.3%)	386 (34.3%)	NS

**Table A5:** Characteristics of the study group by delivery method and Apgar score.

Variable	Group Size (Percentage)	Age—Mean (Standard Deviation)	Vaginal Delivery			Cesarean Section			*p*-Value
			Apgar 0–3	Apgar 4–7	Apgar 8–10	Apgar 0–3	Apgar 4–7	Apgar 8–10	
**Total**	1438 (100.0%)	29.5, 5.0	37 (2.6%)	86 (6.0%)	139 (9.7%	32 (2.2%)	308 (21.4%)	836 (58.1%)	<0.001
**Primiparae**	834 (58.0%)	28.5, 4.9	19 (2.3%)	29 (3.5%)	54 (6.5%)	24 (2.9%)	200 (24.0%)	508 (60.9%)	<0.001
**Multiparae**	604 (42.0%)	30.8, 5.0	18 (3.0%)	57 (9.4%)	85 (14.1%)	8 (1.3%)	108 (17.9%)	328 (54.3%)	<0.001
**MA 18–24**	246 (17.1%)	22.5, 1.7	8 (3.3%)	16 (6.5%)	30 (12.2%)	7 (2.8%)	44 (17.9%)	141 (57.3%)	<0.01
**MA 25–34**	962 (66.9%)	29.3, 2.8	23 (2.4%)	55 (5.7%)	92 (9.6%)	21 (2.2%)	218 (22.7%)	553 (57.5%)	<0.001
**MA > 34**	230 (16.0%)	37.5, 2.4	6 (2.6%)	15 (6.5%)	17 (7.4%)	4 (1.7%)	46 (20.0%)	142 (61.7%)	<0.001
**GA < 28 weeks**	38 (2.6%)	27.5, 3.3	12 (31.6%)	6 (15.8%)	0 (0.0%)	4 (10.5%)	16 (42.1%)	0 (0.0%)	<0.01
**GA 28–32 weeks**	208 (14.5%)	29.5, 5.5	13 (6.2%)	24 (11.5%)	3 (1.4%)	15 (7.2%)	95 (45.7%)	58 (27.9%)	<0.001
**GA 33–37 weeks**	886 (61.6%)	29.5, 4.8	10 (1.1%)	35 (4.0%)	65 (7.3%)	8 (0.9%)	161 (18.2%)	607 (68.5%)	<0.001
**GA 38–43 weeks**	306 (21.3%)	29.5, 5.5	2 (0.7%)	21 (6.9%)	71 (23.2%)	5 (1.6%)	36 (11.8%)	171 (55.9%)	NS
**Twin I**	719 (50.0%)	29.5, 5.0	20 (2.8%)	54 (7.5%)	57 (7.9%)	22 (3.1%)	173 (24.1%)	393 (54.7%)	<0.001
**Twin II**	719 (50.0%)	29.5, 5.0	17 (2.4%)	32 (4.5%)	82 (11.4%)	10 (1.4%)	135 (18.8%)	443 (61.6%)	<0.001
**IVF**	314 (21.8%)	30.7, 4.2	0 (0.0%)	9 (2.9%)	9 (2.9%)	12 (3.8%)	94 (29.9%)	198 (63.1%)	NS
**Spontaneous conception**	1124 (78.2%)	29.1, 5.2	37 (3.3%)	85 (7.6%)	130 (11.6%)	20 (1.8%)	214 (19.0%)	638 (56.8%)	<0.001
**MCDA**	310 (21.6%)	28.9, 5.4	9 (2.9%)	22 (7.1%)	21 (6.8%)	9 (2.9%)	76 (24.5%)	173 (55.8%)	<0.001
**DCDA**	1128 (78.4%)	29.6, 4.9	28 (2.5%)	64 (5.7%)	118 (10.5%)	23 (2.0%)	232 (20.6%)	663 (58.8%)	<0.001
**Weight difference < 15%**	992 (69.0%)	29.5, 5.1	19 (1.9%)	62 (6.2%)	105 (10.6%)	17 (1.7%)	188 (19.0%)	601 (60.6%)	<0.001
**Weight difference** **15–19%**	196 (13.6%)	29.2, 4.7	6 (3.1%)	8 (4.1%)	14 (7.1%)	0 (0.0%)	57 (29.1%)	111 (56.6%)	<0.001
**Weight difference** **20–24%**	80 (5.6%)	29.9, 5.6	2 (2.5%)	11 (13.8%)	9 (11.2%)	3 (3.8%)	13 (16.2%)	42 (52.5%)	<0.05
**Weight difference** **25–30%**	78 (5.4%)	29.9, 5.0	2 (2.6%)	4 (5.1%)	6 (7.7%)	1 (1.3%)	21 (26.9%)	44 (56.4%)	<0.05
**Weight difference** **>30%**	92 (6.4%)	29.0, 4.4	8 (8.7%)	1 (1.1%)	5 (5.4%)	11 (12.0%)	29 (31.5%)	38 (41.3%)	<0.001
**Smaller twin**	719 (50.0%)	29.5, 5.0	19 (2.6%)	45 (6.3%)	67 (9.3%)	21 (2.9%)	146 (20.3%)	421 (58.6%)	<0.001
**Bigger twin**	719 (50.0%)	29.5, 5.0	18 (2.5%)	41 (5.7%)	72 (10.0%)	11 (1.5%)	162 (22.5%)	415 (57.7%)	<0.001

**Table A6:** Characteristics of the study group by birth order and Apgar score.

Variable	Group Size (Percentage)	Age—Mean (Standard Deviation)	Twin I			Twin II			*p*-Value
			Apgar 0–3	Apgar 4–7	Apgar 8–10	Apgar 0–3	Apgar 4–7	Apgar 8–10	
**Total**	1438 (100.0%)	29.5, 5.0	42 (2.9%)	227 (15.8%)	450 (31.3%)	27 (1.9%)	167 (11.6%)	525 (36.5%)	<0.001
**Primiparae**	834 (58.0%)	28.5, 4.9	26 (3.1%)	132 (15.8%)	259 (31.1%)	17 (2.0%)	97 (11.6%)	303 (36.3%)	<0.01
**Multiparae**	604 (42.0%)	30.8, 5.0	16 (2.6%)	95 (15.7%)	191 (31.6%)	10 (1.7%)	70 (11.6%)	222 (36.8%)	<0.05
**MA 18–24**	246 (17.1%)	22.5, 1.7	9 (3.7%)	34 (13.8%)	80 (32.5%)	6 (2.4%)	26 (10.6%)	91 (37.0%)	NS
**MA 25–34**	962 (66.9%)	29.3, 2.8	27 (2.8%)	158 (16.4%)	296 (30.8%)	17 (1.8%)	115 (12.0%)	349 (36.3%)	<0.01
**MA > 34**	230 (16.0%)	37.5, 2.4	6 (2.6%)	35 (15.2%)	74 (32.2%)	4 (1.7%)	26 (11.3%)	85 (37.0%)	NS
**GA < 28 weeks**	38 (2.6%)	27.5, 3.3	8 (21.1%)	11 (28.9%)	0 (0.0%)	8 (21.1%)	11 (28.9%)	0 (0.0%)	NS
**GA 28–32 weeks**	208 (14.5%)	29.5, 5.5	19 (9.1%)	56 (26.9%)	29 (13.9%)	9 (4.3%)	63 (30.3%)	32 (15.4%)	NS
**GA 33–37 weeks**	886 (61.6%)	29.5, 4.8	10 (1.1%)	121 (13.7%)	312 (35.2%)	8 (0.9%)	75 (8.5%)	360 (40.6%)	<0.001
**GA 38–43 weeks**	306 (21.3%)	29.5, 5.5	5 (1.6%)	39 (12.7%)	109 (35.6%)	2 (0.7%)	18 (5.9%)	133 (43.5%)	<0.01
**IVF**	314 (21.8%)	30.7, 4.2	7 (2.2%)	55 (17.5%)	95 (30.3%)	5 (1.6%)	40 (12.7%)	112 (35.7%)	NS
**Spontaneous conception**	1124 (78.2%)	29.1, 5.2	35 (3.1%)	172 (15.3%)	355 (31.6%)	22 (2.0%)	127 (11.3%)	413 (36.7%)	<0.001
**Vaginal delivery**	262 (18.2%)	28.9, 5.3	20 (7.6%)	54 (20.6%)	57 (21.8%)	17 (6.5%)	32 (12.2%)	82 (31.3%)	<0.01
**Cesarean section**	1176 (81.8%)	29.6, 5.0	22 (1.9%)	173 (14.7%)	393 (33.4%)	10 (0.9%)	135 (11.5%)	443 (37.7%)	<0.01
**MCDA**	310 (21.6%)	28.9, 5.4	12 (3.9%)	56 (18.1%)	87 (28.1%)	6 (1.9%)	42 (13.5%)	107 (34.5%)	<0.05
**DCDA**	1128 (78.4%)	29.6, 4.9	30 (2.7%)	171 (15.2%)	363 (32.2%)	21 (1.9%)	125 (11.1%)	418 (37.1%)	<0.01
**Weight difference < 15%**	992 (69.0%)	29.5, 5.1	24 (2.4%)	147 (14.8%)	325 (32.8%)	12 (1.2%)	103 (10.4%)	381 (38.4%)	<0.001
**Weight difference** **15–19%**	196 (13.6%)	29.2, 4.7	3 (1.5%)	40 (20.4%)	55 (28.1%)	3 (1.5%)	25 (12.8%)	70 (35.7%)	NS
**Weight difference** **20–24%**	80 (5.6%)	29.9, 5.6	3 (3.8%)	12 (15.0%)	25 (31.2%)	2 (2.5%)	12 (15.0%)	26 (32.5%)	NS
**Weight difference** **25–30%**	78 (5.4%)	29.9, 5.0	2 (2.6%)	11 (14.1%)	26 (33.3%)	1 (1.3%)	14 (17.9%)	24 (30.8%)	NS
**Weight difference** **>30%**	92 (6.4%)	29.0, 4.4	10 (10.9%)	17 (18.5%)	19 (20.7%)	9 (9.8%)	13 (14.1%)	24 (26.1%)	NS
**Smaller twin**	719 (50.0%)	29.5, 5.0	20 (2.8%)	90 (12.5%)	170 (23.6%)	20 (2.8%)	101 (14.0%)	318 (44.2%)	<0.01
**Bigger twin**	719 (50.0%)	29.5, 5.0	22 (3.1%)	137 (19.1%)	280 (38.9%)	7 (1.0%)	66 (9.2%)	207 (28.8%)	<0.05

A summary of the results presented in Table A1, Table A2, Table A3, Table A4, Table A5 and Table A6 is provided in Table A7.

**Table A7:** Summary of *p*-value and conclusions across study groups.

Variable	*p*-Value	Conclusion
Mode of conception	NS	Method of conception does not significantly influence the postnatal condition of twins assessed by the 5-minute Apgar score.
Chorionicity and amnionicity	NS	Chorionicity and amnionicity does not significantly influence the postnatal condition of twins assessed by the 5-minute Apgar score.
Weight difference (percentage between twins)	*p* < 0.05	Twins with weight difference < 15% proportionally more likely received a good 5–minute Apgar score.
Weight difference (being bigger or smaller twin)	NS	Being a bigger or smaller twin does not significantly influence the postnatal condition of twins assessed by the 5-minute Apgar score.
Delivery method	*p* < 0.05	Twins born by a cesarean section proportionally more likely received a good 5–minute Apgar score.
Birth order	*p* < 0.05	Second-born twins proportionally more likely received a good 5-minute Apgar score.

## References

[1] Rebar R.W. (2013). What are the risks of the assisted reproductive technologies (ART) and how can they be minimized?. Reprod. Med. Biol..

[2] Caserta D., Bordi G., Stegagno M., Filippini F., Podagrosi M., Roselli D., Moscarini M. (2014). Maternal and perinatal outcomes in spontaneous versus assisted conception twin pregnancies. Eur. J. Obstet. Gynecol. Reprod. Biol..

[3] Marleen S., Kodithuwakku W., Nandasena R., Mohideen S., Allotey J., Fernández-García S., Gaetano-Gil A., Ruiz-Calvo G., Aquilina J., Khalil A. (2024). Maternal and perinatal outcomes in twin pregnancies following assisted reproduction: A systematic review and meta-analysis involving 802 462 pregnancies. Hum. Reprod. Update.

[4] Reimundo P., Gutiérrez Romero J.M., Rodríguez Pérez T., Veiga E. (2021). Single-embryo transfer: A key strategy to reduce the risk for multiple pregnancy in assisted human reproduction. Adv. Lab. Med..

[5] Ochsenbein-Kölble N. (2021). Twin pregnancies. Ultraschall Med..

[6] Tsakiridis I., Giouleka S., Mamopoulos A., Athanasiadis A., Dagklis T. (2020). Management of twin pregnancies: A comparative review of national and international guidelines. Obstet. Gynecol. Surv..

[7] Duy Anh N., Thu Ha N.T., Khac Toan N., Tuan Dat D., Huyen Thuong P.T., Tra Giang D.T., Duc T.A., Anh B.X., Ha N.M., Duc N.M. (2022). Obstetric and Perinatal Outcomes of Dichorionic-Diamniotic Twin Pregnancies Conceived by IVF/ICSI Compared with Those Conceived Spontaneously. Clin. Ter..

[8] Jordan B.K., Bernard L., Segel S., Go M.D., Schilling D., McEvoy C.T. (2023). Premature monochorionic monoamniotic twins have lower lung compliance at birth than matched dichorionic diamniotic twins. J. Neonatal Perinat. Med..

[9] Zhu J., Zhang J., Wu Y., Gao L., Zhao X., Cheng W., Wang Y. (2023). Intertwin growth discordance throughout gestation and hypertensive disorders of pregnancy. Am. J. Obstet. Gynecol..

[10] Chew L.C., Osuchukwu O.O., Reed D.J., Verma R.P. (2025). Fetal Growth Restriction. StatPearls.

[11] Helmerhorst F.M., Perquin D.A.M., Donker D., Keirse M.J.N.C. (2004). Perinatal outcome of singletons and twins after assisted conception: A systematic review of controlled studies. BMJ.

[12] Barrett J.F.R. (2004). Delivery of the term twin. Best. Pract. Res. Clin. Obstet. Gynaecol..

[13] Konar H., Sarkar M., Paul J. (2016). Perinatal outcome of the second twin at a tertiary care center in India. J. Obstet. Gynaecol. India.

[14] Di Mascio D., Acharya G., Khalil A., Odibo A., Prefumo F., Liberati M., Buca D., Manzoli L., Flacco M.E., Brunelli R. (2019). Birthweight discordance and neonatal morbidity in twin pregnancies: A systematic review and meta-analysis. Acta Obstet. Gynecol. Scand..

[15] Shah J.S., Nasab S.H., Chappell N., Chen H.-Y., Schutt A., Mendez-Figueroa H. (2018). Neonatal outcomes among twins stratified by method of conception: Secondary analysis of maternal fetal medicine (MFMU) network database. J. Assist. Reprod. Genet..

[16] Andrijasevic S., Dotlic J., Aksam S., Micic J., Terzic M. (2014). Impact of conception method on twin pregnancy course and outcome. Geburtshilfe Frauenheilkd..

[17] Lin L., Yao T., Liao Q., Liu J., Huang L., Zheng L. (2024). Neonatal outcomes among twins born through assisted reproduction, compared to those born naturally. Medicine.

[18] Özçil M.D. (2021). Comparison of Feto-maternal Effects of Twin Pregnancies and Twin Pregnancies Caused by Assisted Reproductive Technology. J. Acad. Res. Med..

[19] Nazneen S., Kandasamy V., Shinde R.V. (2024). A comparative study of spontaneously conceived twin pregnancies vs. twins conceived by assisted reproductive techniques and their maternal and perinatal outcomes: A prospective observational study. Int. J. Reprod. Contracept. Obstet. Gynecol..

[20] Henningsen A.K., Pinborg A. (2014). Birth and perinatal outcomes and complications for babies conceived following ART. Semin. Fetal Neonatal Med..

[21] Pelkonen S., Koivunen R., Gissler M., Nuojua-Huttunen S., Suikkari A.M., Hydén-Granskog C., Martikainen H., Tiitinen A., Hartikainen A.-L. (2010). Perinatal outcome of children born after frozen and fresh embryo transfer: The Finnish cohort study 1995–2006. Hum Reprod..

[22] Luke B., Brown M.B., Missmer S.A., Bukulmez O., Leach R., Stern J.E., Society for Assisted Reproductive Technology writing group (2011). The effect of increasing obesity on the response to and outcome of assisted reproductive technology: A national study. Fertil. Steril..

[23] Rashid D., Alalaf S. (2020). Maternal and perinatal outcomes in twin pregnancies conceived spontaneously and by assisted reproductive techniques: Cross-sectional study. East. Mediterr. Health J..

[24] Coutinho Nunes F., Domingues A.P., Vide Tavares M., Belo A., Ferreira C., Fonseca E., Moura P. (2016). Monochorionic versus dichorionic twins: Are obstetric outcomes always different?. J. Obstet. Gynaecol..

[25] Rissanen A.-R.S., Gissler M., Nupponen I.K., Nuutila M.E., Jernman R.M. (2022). Perinatal outcome of dichorionic and monochorionic-diamniotic Finnish twins: A historical cohort study. Acta Obstet. Gynecol. Scand..

[26] Wandel L., Abele H., Pauluschke-Fröhlich J., Kagan K.O., Brucker S., Rall K. (2022). Mode of birth in monochorionic versus dichorionic twin pregnancies: A retrospective study from a large tertiary centre in Germany. BMC Pregnancy Childb..

[27] Miranda F., Teixeira A., Castro L., Carvalho C., Lopes L. (2024). Neonatal outcome of second-born twins: A 15-year retrospective study. J. Pediatr. Neonatal Individ. Med..

[28] Appleton C., Pinto L., Centeno M., Clode N., Cardoso C., Graça L.M. (2007). Near term twin pregnancy: Clinical relevance of weight discordance at birth. J. Perinat. Med..

[29] Yang L., Zhou Y., Qiu J., Lin N., Gu N., Dai Y. (2024). Birth weight discordance and adverse neonatal outcomes in appropriately grown premature twins. Heliyon.

[30] Yum S.K., Lee J.H. (2023). Role of birthweight discordance in preterm twins’ outcomes in the Korean neonatal network. Pediatr. Neonatol..

[31] D’Antonio F., Odibo A.O., Prefumo F., Khalil A., Buca D., Flacco M.E., Liberati M., Manzoli L., Acharya G. (2018). Weight discordance and perinatal mortality in twin pregnancy: Systematic review and meta-analysis. Ultrasound Obstet. Gynecol..

[32] Bagchi S., Salihu H.M. (2006). Birth weight discordance in multiple gestations: Occurrence and outcomes. J. Obstet. Gynaecol..

[33] Wen S.W., Fung K.F.K., Huang L., Demissie K., Joseph K.S., Allen A.C., Kramer M.S., Fetal N.F.T., Infant Health Study Group of the Canadian Perinatal Surveillance System (2005). Fetal and neonatal mortality among twin gestations in a Canadian population: The effect of intrapair birthweight discordance. Am. J. Perinatol..

[34] Branum A.M., Schoendorf K.C. (2003). The effect of birth weight discordance on twin neonatal mortality. Obstet. Gynecol..

[35] Demissie K., Ananth C.V., Martin J., Hanley M.L., MacDorman M.F., Rhoads G.G. (2002). Fetal and neonatal mortality among twin gestations in the United States: The role of intrapair birth weight discordance. Obstet. Gynecol..

[36] Florjański J.S., Homola W., Fuchs T., Pawłosek A., Kasperski M. (2019). Postnatal condition of the second twin in respect to mode of delivery, chorionicity and type of fetal growth. Adv. Clin. Exp. Med..

[37] Axelsdóttir Í., Ajne G. (2019). Short-term outcome of the second twin during vaginal delivery is dependent on delivery time interval but not chorionicity. J. Obstet. Gynaecol..

[38] Rahman R.A., Mohammed Nawi A., Ishak S., Balaraman K., Abu M.A., Abd Azman S.H., Kalok A.H., Ismail N.A.M., Mahdy Z.A., Ahmad S. (2024). Second twin outcome at birth: Retrospective analysis in a single tertiary centre in Malaysia. J. Perinat. Med..

[39] Cukierman R., Heland S., Palmer K., Neil P., da Silva Costa F., Rolnik D.L. (2019). Inter-twin delivery interval, short-term perinatal outcomes and risk of caesarean for the second twin. Aust. N. Z. J. Obstet. Gynaecol..

[40] Tal A., Peretz H., Garmi G., Zafran N., Romano S., Salim R. (2018). Effect of inter-twin delivery interval on umbilical artery pH and Apgar score in the second twin. Birth.

[41] Algeri P., Callegari C., Mastrolia S.A., Brienza L., Vaglio Tessitore I., Paterlini G., Incerti M., Cozzolino S., Vergani P. (2019). What is the effect of intertwin delivery interval on the outcome of the second twin delivered vaginally?. J. Matern. Fetal Neonatal Med..

[42] Algeri P., Callegari C., Bernasconi D.P., Incerti M., Cozzolino S., Paterlini G., Mastrolia S.A., Pellizzoni F., Vergani P. (2019). Neonatal hypoxia of the second twin after vaginal delivery of the first twin: What matters?. J. Matern. Fetal Neonatal Med..

[43] Benito M., De Bonrostro C., Agustín A., Roca M., Campillos J.M., Castán S. (2019). Impact of intertwin interval on short-term neonatal outcomes of the second twin in dichorionic pregnancies with vaginal delivery. Int. J. Gynaecol. Obstet..

[44] Stein W., Misselwitz B., Schmidt S. (2008). Twin-to-twin delivery time interval: Influencing factors and effect on short-term outcome of the second twin. Acta Obstet. Gynecol. Scand..

[45] Lindroos L., Elfvin A., Ladfors L., Wennerholm U.B. (2018). The effect of twin-to-twin delivery time intervals on neonatal outcome for second twins. BMC Pregnancy Childb..

[46] Erdemoglu E., Mungan T., Tapisiz O.L., Ustunyurt E., Caglar E. (2003). Effect of inter-twin delivery time on Apgar scores of the second twin. Aust. N. Z. J. Obstet. Gynaecol..

[47] Mok S.L., Lo T.K. (2022). Vaginal delivery of second twins: Factors predictive of failure and adverse perinatal outcomes. Hong Kong Med. J..

[48] Chibwesha C.J., Zanolini A., Smid M., Vwalika B., Phiri Kasaro M., Mwanahamuntu M., Stringer J.S., Stringer E.M. (2016). Predictors and outcomes of low birth weight in Lusaka, Zambia. Int. J. Gynaecol. Obstet..

[49] Thorngren-Jerneck K., Herbst A. (2001). Low 5-minute Apgar score: A population-based register study of 1 million term births. Obstet. Gynecol..

[50] Eller D.P., VanDorsten J.P. (1995). Route of delivery for the breech presentation: A conundrum. Am. J. Obstet. Gynecol..

[51] Jhaveri R.R., Nadkarni T.K. (2016). Perinatal Outcome of Second Twin with Respect to Mode of Delivery: An Observational Study. J. Clin. Diagn. Res..

[52] Adam C., Allen A.C., Baskett T.F. (1991). Twin delivery: Influence of the presentation and method of delivery on the second twin. Am. J. Obstet. Gynecol..

[53] Armson B.A., O’Connell C., Persad V., Joseph K.S., Young D.C., Baskett T.F. (2006). Determinants of perinatal mortality and serious neonatal morbidity in the second twin. Obstet. Gynecol..

[54] Ylilehto E., Palomäki O., Huhtala H., Uotila J. (2017). Term twin birth—Impact of mode of delivery on outcome. Acta Obstet. Gynecol. Scand..

[55] Rossi A.C., Mullin P.M., Chmait R.H. (2011). Neonatal outcomes of twins according to birth order, presentation and mode of delivery: A systematic review and meta-analysis. BJOG Int. J. Obstet. Gynaecol..

[56] Barrett J.F.R., Hannah M.E., Hutton E.K., Willan A.R., Allen A.C., Armson B.A., Gafni A., Joseph K., Mason D., Ohlsson A. (2013). A randomized trial of planned cesarean or vaginal delivery for twin pregnancy. N. Engl. J. Med..

[57] Sadeh-Mestechkin D., Daykan Y., Bustan M., Markovitch O., Shechter-Maor G., Biron-Shental T. (2018). Trial of vaginal delivery for twins—Is it safe? A single center experience. J. Matern. Fetal Neonatal Med..

[58] Hofmeyr G.J., Barrett J.F., Crowther C.A. (2011). Planned caesarean section for women with a twin pregnancy. Cochrane Database Syst. Rev..

